# Secondary solid malignancies in long-term survivors after total body irradiation

**DOI:** 10.1186/s13014-024-02520-8

**Published:** 2024-09-17

**Authors:** Isabella Gruber, Daniel Wolff, Oliver Koelbl

**Affiliations:** 1 Department of Radiation Oncology, University Hospital Regensburg, Franz-Josef-Strauß Allee 11, Regensburg, Germany; 2 Department of Internal Medicine III, University Hospital Regensburg, Franz-Josef-Strauß Allee 11, Regensburg, Germany

**Keywords:** Total body irradiation, Low-dose radiotherapy, Secondary solid malignancies, Allogeneic hematopoietic stem cell transplantation, Carcinogenesis, Acute myeloid leukemia

## Abstract

**Background:**

Total body irradiation (TBI)-based allogeneic hematopoietic stem cell transplantation (allo-HSCT) is a curative treatment for selected patients with acute myeloid leukemia (AML). Yet, secondary malignancies contribute to long-term morbidity and mortality with TBI potentially influencing these risks.

**Methods:**

This retrospective study analyzed the cumulative incidences of secondary solid malignancies and precancerous lesions of 89 consecutive AML patients after TBI-based conditioning before 1st allo-HSCT between 2000 and 2016. TBI was performed with an average dose rate of 4 cGy/min and a twice-daily fractionation. Cause-specific hazard models analyzed risk factors for secondary malignancies/precancerous lesions and the competing risks of dying before developing secondary malignancies/precancerous lesions.

**Results:**

The median patient age at TBI was 42.5 years (interquartile range, 32.5–51.2), while the median follow-up was 15.2 years (interquartile range, 13.0-18.2). Most patients received a myeloablative conditioning (MAC) containing 8 Gy (*n* = 47) and 12 Gy TBI (*n* = 11). Reduced-intensity regimens (RIC, 4 Gy TBI) were applied in 31 patients. Of note, patients receiving RIC were older than patients receiving MAC. The most common cancer types were non-squamous cell carcinomas (*n* = 14) after exclusion of a patient diagnosed with sarcoma within less than a year after TBI. The cumulative incidences of secondary malignancies and precancerous lesions were 8% (95%CI, 4–16), 14% (95%CI, 7–23), and 17% (95%CI, 9–27) at 10, 15 and 20 years, while the cumulative incidences of premature deaths were 59% (95%CI, 48–69), 59% (95%CI, 48–69), and 64% (95%CI, 49–76). In multivariate analyses, higher patient age at TBI was associated with lower rates of secondary malignancies/precancerous lesions, while higher patient age translated into a trend towards premature deaths (before patients could develop malignancies). Higher TBI doses, mainly applied in younger patients, translated into lower rates of secondary malignancies/precancerous lesions while lacking associations with mortality. Chronic GVHD requiring systemic immunosuppression was associated with premature deaths.

**Conclusions:**

Although this study indicates an inverse relationship between TBI doses applied and treatment-related malignancies, confounding by competing risks is present. The age dependency may be explained by the fact that older patients had a lower life expectancy independent of malignancies, illustrating the pitfalls of competing risks.

**Trial registration:**

The study was retrospectively registered.

## Background

Allogeneic hematopoietic stem cell transplantation (allo-HSCT) applying TBI and non-TBI-based conditioning regimens is a curative treatment option for selected patients with acute myeloid leukemia (AML). Yet, secondary malignancies contribute to long-term morbidity and mortality with conditioning regimens potentially modulating these risks [1]. Myeloablative conditioning (MAC) containing higher doses of TBI (8–12 Gy) is usually applied in fit and young patients (< 50 years of age) [2, 3]. Reduced-intensity conditioning (RIC) combining lower doses of chemotherapy and/or TBI (doses ranging from ≥ 4 Gy to < 8 Gy) is used particularly in patients ineligible for myeloablative conditioning to reduce transplant-related complications while maintaining efficacy [2, 4]. Both recipients of TBI and non-TBI-based conditioning regimens are at risk of developing secondary malignancies after allo-HSCT [5, 6]. Literature indicates that TBI is associated with basal cell carcinomas, and breast, thyroid, and brain cancers occurring with a long delay after allo-HSCT [7–9]. In contrast, chronic graft-versus-host disease (cGVHD)-related squamous cell carcinomas (SCCs) of the skin and oropharynx seem to occur after shorter latency [5]. Cumulative incidences of secondary solid malignancies after allo-HSCT as reported in the literature vary depending on statistical methodologies, patient population, conditioning regimens, and cGVHD and are as high as 13.5% at 15 years [10] and 22% at 30 years [11]. Yet, data are sparse regarding incidences of secondary solid malignancies after allo-HSCT applying modern and uniform TBI techniques. In this retrospective study, we, therefore, estimated the cumulative incidences of secondary solid malignancies and precancerous lesions in the presence of the competing risks of premature mortality after applying a standardized fractionated TBI technique for AML patients and 1st allo-HSCT. Cause-specific hazard models analyzed associations between transplant characteristics and the rates of secondary solid malignancies/precancerous lesions including the competing risks of premature death before developing malignancies.

## Methods

### Data collection

We retrospectively analyzed the cumulative incidences of secondary solid malignancies and precancerous lesions in adult patients with primary or secondary AML receiving TBI as part of the conditioning regimen before 1st allo-HSCT at the Departments of Radiation Oncology and Hematology of the University Hospital Regensburg between 01/2000 and 10/2016. Patients conditioned with TBI after 10/2016 were not analyzed to ensure sufficient follow-up. As patients conditioned with non-TBI-based regimens were older, this study didn´t analyze non-TBI-based regimens to prevent an age bias [12]. Secondary solid malignancies were subdivided into squamous cell carcinomas (SCCs) and non-squamous cell carcinomas (non-SCCs). Clinical data were extracted from the medical charts of the Departments of Radiation Oncology and Hematology of the University Hospital Regensburg. Transplantation variables included patient age at the time of TBI, conditioning regimens (RIC, 4 Gy TBI; MAC, 8 Gy and 12 Gy TBI), sex, diagnosis, Karnofsky performance score (KPS), hematopoietic cell transplantation-comorbidity index (HCT-CI) [13], 2017 European LeukemiaNet (ELN) genetic risk stratification [14], disease status before TBI, stem cell source, recipient and donor characteristics and GVHD prophylaxis. Graft-versus-host disease prophylaxis and conditioning regimens were dependent on the patient´s age, comorbidities and disease risk. The use of antithymocyte globulin (ATG) as part of GVHD prophylaxis was standard in unrelated donor transplantation and at the discretion of the physicians in sibling donor transplantation. Variables related to outcome were the cumulative incidences of secondary solid malignancies and precancerous lesions, relapse, non-relapse mortality (NRM), cGVHD (requiring systemic immunosuppression) and grade II-IV aGVHD. All patients received screening examinations for cutaneous malignancies before TBI. The screening of secondary malignancies after allo-HSCT included annual physical examinations, which encompassed examinations of the thyroid glands, skin, oropharynx and oral cavity. Patients suffering from cGVHD and those at high risk for developing cancers of the oropharynx and oral cavity were examined every 6 months. Gynecological and urological screenings for secondary solid malignancies were performed annually. Data closing was in October 2023. The local Ethics Board of the University of Regensburg approved this study (approval number, 20-1810-101).

### TBI

Details of our TBI technique were previously published [15, 16]. We used Siemens Primus linear accelerators (Siemens Medical Systems, Inc., Concord, CA) and linear accelerators of type Elekta Synergy ™ with an Agility ™ head (Elekta Ltd, Crawley, UK) during the study period. All patients received rotational arcs with 6 megavoltage (MV) photon beams. We used a twice-daily fractionation and a minimum of 6 h between fractions. The average dose rate to the body was 4 cGy/min. Two individual lung shields of MCP96 with calculated thickness were designed if doses exceeded 8 Gy to reduce the total dose to the center of the lung to 7 Gy. Areas of the chest wall shielded by the lung blocks were irradiated once a day with electron beams to achieve full doses to the thoracic walls [15].

### Definitions and statistical endpoints

Competing risks are common in survival data describing an event (e.g. premature deaths in patients before they could develop secondary malignancies) that prevents the event of interest, e.g. development of secondary malignancies [17, 18]. We used, therefore, the cumulative incidence function (CIF) to describe the cumulative incidences of secondary solid malignancies and precancerous lesions accounting for the competing risks of premature deaths among patients who did not develop secondary solid malignancies/precancerous lesions. Cause-specific hazard models analyzed the impact of pre-transplantation variables on the rates of secondary malignancies/precancerous lesions and the competing risks of premature deaths. Risk factors evaluated were patient age at TBI, TBI dose, ATG as part of GVHD prophylaxis, and cGVHD requiring immunosuppressive therapy. Age at TBI and TBI doses were analyzed as continuous variables. The impact of cGVHD changing value throughout the observation period on the rates of secondary solid malignancies/precancerous lesions was analyzed by applying a counting process format [19], while patients entered the risk set at the age of TBI. Secondary solid malignancies included solid cancers of any site and histology after TBI while excluding post-transplant lymphoproliferative disorders. The study recorded the times to the first secondary malignancy for patients developing ≥ 2 secondary malignancies. Acute GVHD and cGVHD were defined according to described standard criteria [20, 21]. Acute GVHD was classified as clinically significant at grade II-IV aGVHD. For the cumulative incidences of cGVHD requiring systemic immunosuppressive therapy, relapse or death without prior cGVHD was counted as a competing event. We captured non-relapse mortality (NRM) and relapse of AML. NRM was defined as deaths from any cause in the absence of prior relapse of the initial AML, with relapse considered a competing event. Relapse was defined as manifest hematologic relapse requiring treatment.

### Statistical analysis

This study presents continuous variables as median and interquartile range (IQR) and categorical variables as absolute and relative frequencies. We used the cumulative incidence function (CIF) to estimate cumulative incidences of secondary malignancies and precancerous lesions, relapse, NRM, and cGVHD in the presence of competing risks [18]. CIF were compared using Gray´s test. The effects of transplantation variables on the rates of secondary malignancies/precancerous lesions and the competing risks of premature deaths were estimated with cause-specific hazard (CSH) analyses treating all other events as censored. The proportional hazards assumption of the CSH model was tested using Schoenfeld residuals. Hazard Ratio (HR) and 95% confidence intervals (95% CI) were presented as effect estimates. Median follow-up time was estimated using the reverse Kaplan-Meier method. All *P*-values were two-sided, and *P*-values < 0.05 were considered significant. Statistical analysis was performed using R, version 4.3.2 (R Core Team. R: A language for statistical computing. 2014. The R Foundation for Statistical Computing, Vienna, Austria) and SPSS 26.0 (SPSS Inc., Chicago, IL, USA).

## Results

### Patient and transplantation characteristics

Eighty-nine patients received TBI-based conditioning before 1st allo-HSCT. Table 1 summarizes transplant characteristics. Median patient age at TBI was 42.5 years (IQR, 32.5–51.2). The median follow-up time was 15.2 years (IQR, 13.0-18.2).

**Table 1:** Patients characteristics (*n* = 89)

Characteristics	value
**Follow-up**, years, median (IQR)	15.2 (13.0-18.2)
**Patient age**, median (IQR)	42.5 (32.5–51.2)
**Sex**, n (%)	
male female	56 (62.9%) 33 (37.1%)
**Diagnosis**, n (%)	
de novo acute myeloid leukemia secondary acute myeloid leukemia	68 (76.4%) 21 (23.6%)
**Karnofsky performance score**	
< 80 ≥ 80	8 (9.0%) 81 (91.0%)
**Hematopoietic cell transplantation-comorbidity index (HCT-CI)**, n (%)	
0 1–2 ≥ 3	40 (44.9%) 34 (38.2%) 15 (16.9%)
**2017 ELN genetic risk stratification**, n (%)	
favorable intermediate adverse	13 (14.6%) 36 (40.4%) 40 (44.9%)
**Disease status at allo-HSCT**, n (%)	
First complete remission, CR1 CR2, first partial remission, PR1 > CR2, refractory, active disease	31 (34.8%) 32 (36.0%) 26 (29.2%)
**Donor type**, n (%)	
matched sibling donor matched unrelated donor mismatched unrelated donor	32 (36.0%) 50 (56.2%) 7 (7.9%)
**Stem cell source**, n (%)	
peripheral blood bone marrow	82 (92.1%) 7 (7.9%)
**Conditioning regimens**, n (%)	
reduced intensity (4 Gy TBI) myeloablative intensity (8 Gy TBI, 12 Gy TBI)	31 (34.8%) 58 (65.2%)
**Donor age**, years, median (IQR)	38.0 (30.5–44.0)
**Female donors to male recipients**, n (%)	
yes no	14 (15.7%) 75 (84.3%)
**Graftversushost disease prophylaxis**, n (%)	
Cyclosporine, MTX Cyclosporine, MMF Post-transplant Cyclophosphamide, Tacrolimus, MMF	57 (64.0%) 30 (33.7%) 2 (2.2%)
Antithymocyte globulin (ATG) *, n (%)	
yes no	61 (68.5%) 28 (31.5%)

*CMV*, cytomegalovirus; *ELN*, European LeukemiaNet; * *ATG* was part of graft-versus-host disease prophylaxis; *MTX*, Methotrexate; *MMF*, Mycophenolate Mofetil

Most patients (*n* = 47) received a myeloablative conditioning (MAC) regimen containing 8 Gy TBI. Reduced-intensity conditioning (RIC) regimens comprising 4 Gy TBI were applied in 31 patients, while eleven patients received MAC with 12 Gy TBI (Table 2). The median age of patients receiving RIC (4 Gy) and MAC (8–12 Gy) was 50.7 years (IQR, 41.0–59.0) and 38.9 years (IQR, 29.7–45.1). This difference was statistically significant (*P* < 0.001). ELN genetic risk classification was similar between RIC and MAC groups (*P* = 0.443), while the distribution of disease status at allo-HSCT was different (*P* < 0.001). Fifteen patients receiving RIC were in > 2nd complete remission (including refractory AML), while 11 patients receiving MAC were in > 2nd complete remission (including refractory AML). Most patients receiving MAC were in first complete remission (*n* = 28), while only 3 patients receiving RIC were in first complete remission at allo-HSCT. PR1 and CR2 were present in 13 patients receiving RIC and 19 patients receiving MAC.

**Table 2:** Conditioning regimens before allo-HSCT (*n* = 89)

Regimens	*n* (%)
**TBI 8 Gy**,** Cyclophosphamide**,** Fludarabine** (myeloablative conditioning) 8 Gy TBI (four 2 Gy doses on two consecutive days, d -8, d -7), Cyclophosphamide 2 × 60 mg/kg (d -4, d -3), Fludarabine 3 × 30 mg/m² (d -6, d -5, d -4)	42 (47.2%)
**FLAMSA-RIC**,** TBI 4 Gy**,** Cyclophosphamide** (reduced-intensity conditioning) FLAMSA regimen (d -12 to d -9), Fludarabine 4 × 30 mg/m², HD-Ara-C 4 × 2000 mg/m², Amsacrine 4 × 100 mg/m². Reduced intensity conditioning regimen after 3 days of rest: 4 Gy TBI on d -5 (two 2 Gy doses), Cyclophosphamide (2 × 40 mg/kg for MRD or 2 × 60 mg/kg for MUD, MMRD or MMUD) on d -4 to d -3, Antithymocyte globulin (ATG) 10 mg/kg for MRD or 20 mg/kg for MUD, MMRD, MMUD from d -4 to d -2, prophylactic donor lymphocyte infusions at day + 120 or 30 days after discontinuation of immunosuppression, 1–5 × 10^6^ CD3^+^cells/kg	31 (34.8%)
**TBI 12 Gy**,** Cyclophosphamide** (myeloablative conditioning) 12 Gy TBI (six 2 Gy doses, on three consecutive days, d -7 to d -5), Cyclophosphamide 2 × 60 mg/kg on 2 consecutive days (d -4, d -3)	11 (12.4%)
**TBI 8 Gy**,** Fludarabine** (myeloablative conditioning) 8 Gy TBI (four 2 Gy doses on 2 consecutive days, d -5 and d -4), Fludarabine 4 × 30 mg/m² (d -5 to d -2)	5 (5.6%)

*MRD*, matched related donor; *MUD*, matched unrelated donor; *MMRD*, mismatched related donor; *MMUD*, mismatched unrelated donor

### Chronic graft-versus-host disease

Two-year and 5-year cumulative incidences of cGVHD requiring systemic immunosuppression were 33% (95%CI, 23–42) and 36% (95%CI, 26–46). Twenty-nine patients had a history of grade II-IV aGVHD without cGVHD (requiring systemic immunosuppression), while 26 patients had neither aGVHD nor cGVHD. Eighteen patients had a history of aGVHD and cGVHD, while 16 patients had a history of cGVHD without prior aGVHD. Severe cGVHD was the most frequent maximum grade of cGVHD (*n* = 17) while twelve patients had moderate cGVHD, and five patients had mild cGVHD. Most patients had three or more cGVHD organ sites (median 3, IQR, 2–4). The most common sites of cGVHD in patients suffering from cGVHD were the skin (*n* = 24), oral mucosa (*n* = 20), eyes (*n* = 14), and liver (*n* = 11). The cumulative incidences of cGVHD (requiring systemic immunosuppression) in patients receiving RIC and MAC were similar over the entire follow-up period (*P* = 0.91). Two-year and 5-year cumulative incidences of cGVHD were 35% (95%CI, 19–53) and 39% (95%CI, 21–56) in patients receiving RIC, while patients receiving MAC showed 2-year and 5-year cumulative incidences of cGVHD of 31% (95%CI, 20–43) and 34% (95%CI, 22–47), respectively.

### Secondary malignancies

Table 3 shows details of all secondary solid malignancies and precancerous lesions in 89 patients. Patient no. 290 was diagnosed with a pleomorphic undifferentiated sarcoma which appeared within less than a year after TBI. It was assumed that the sarcoma was present at the time of allo-HSCT. Therefore, this patient was excluded from further analyses resulting in 88 patients.

**Table 3:** Secondary solid malignancies and precancerous lesions after total body irradiation-based conditioning (*n* = 89)

No.	Secondary malignancies (SMs), precancerous lesions	TBI dose	Years from TBI to SMs, † death due to SMs	Age at TBI	Sex	Chronic GVHD requiring systemic immunosuppression, organs of involvement	Smoker
	**Non-squamous cell carcinomas (non-SCCs) in 8 patients**						
419	Mucoepidermoid cancer, lower lip, T1 G1 R0	TBI 4 Gy	11.69	26.2	male	yes, skin. liver, joints	no
266	Medullary thyroid cancer, pT1a pN0 L0 V0 R0	TBI 4 Gy	5.33	40.2	male	yes, skin, oral mucosa, eyes	no
266	Cutaneous basal cell carcinoma, face	TBI 4 Gy	5.33	40.2	male	yes, skin, oral mucosa, eyes	no
19	Mucoepidermoid carcinoma, major salivary gland, pT2 pN1 cM0	TBI 8 Gy	9.63	22.8	male	yes, oral mucosa, eyes, gastrointestinal, liver	no
240	Cutaneous basal cell carcinoma, neck	TBI 8 Gy	12.73	45.8	male	no	yes
240	Cutaneous basal cell carcinoma, face	TBI 8 Gy	12.73	45.8	male	no	yes
290	Pleomorphic undifferentiated sarcoma, pT3 pN1 cM0 R1 *	TBI 8 Gy	0.88, † death after 0.74 years	51.6	female	yes, gastrointestinal	no
117	Papillary thyroid cancer, pT1a L0 V0 R0	TBI 4 Gy	6.02	19.4	male	yes, skin, eyes	no
96	Prostate cancer, adenocarcinoma	TBI 4 Gy	12.45, † death after 3.55 years	60.2	male	no	no
96	Cutaneous basal cell carcinoma, face	TBI 4 Gy	16.0	60.2	male	no	no
307	Cutaneous basal cell carcinoma, face	TBI 8 Gy	14.5	38.6	female	yes, skin	no
307	Cutaneous basal cell carcinoma, face	TBI 8 Gy	14.5	38.6	female	yes, skin	no
307	Cutaneous basal cell carcinoma, face	TBI 8 Gy	15.2	38.6	female	yes, skin	no
307	Cutaneous basal cell carcinoma, face	TBI 8 Gy	16.7	38.6	female	yes, skin	no
307	Cutaneous eccine carcinoma, face	TBI 8 Gy	18.2	38.6	female	yes, skin	no
	**Squamous cell carcinomas (SCCs) in 3 patients**						
428	Cutaneous squamous cell carcinoma, face	TBI 8 Gy	3.20	52.7	male	yes, skin	no
428	Cutaneous squamous cell carcinoma, face	TBI 8 Gy	3.50	52.7	male	yes, skin	no
432	Cutaneous squamous cell carcinoma, face	TBI 8 Gy	16.98	22.9	male	no	yes
9	Squamous cell carcinoma, lip, pT1 cN0 cM0	TBI 8 Gy	9.40	20.3	male	yes, skin, oral mucosa, CNS, gastrointestinal	no
	**Precancerous lesions**,** carcinomas in situ in 3 patients**						
33	Severe intraepithelial neoplasia of the vagina (VIN3)	TBI 8 Gy	7.41	25.1	female	yes, skin, oral mucosa, liver, CNS, eyes, vaginal	no
307	Cutaneous carcinoma in situ, ear	TBI 8 Gy	18.22	38.6	female	yes, skin	no
412	Cutaneous carcinoma in situ, skin	TBI 4 Gy	3.79	48.6	male	yes, skin, oral mucosa, lung, eyes	no

* It was assumed that the pleomorphic undifferentiated sarcoma which appeared within less than a year after TBI was present at the time of allo-HSCT. Therefore, this patient was excluded from further analyses

The cumulative incidences of secondary solid malignancies and precancerous lesions were 8% (95%CI, 4–16), 14% (95%CI, 7–23) and 17% (95%CI, 9–27) at 10, 15 and 20 years, while the cumulative incidences of premature deaths were 59% (95%CI, 48–69), 59% (95%CI, 48–69), and 64% (95%CI, 49–76), respectively. Seven patients developed at least one non-SCC. The most common non-SCCs were cutaneous basal cell carcinomas of the face, while two male patients developed thyroid cancers. The mean time from allo-HSCT to the development of the first non-SCC was 10 years (95%CI, 7–14). Four secondary SCCs occurred in 3 patients. Cutaneous SCCs were the most frequent SCCs. The mean time from allo-HSCT to the first SCC was 10 years (95%CI, -7-27).

**Fig. 1:**
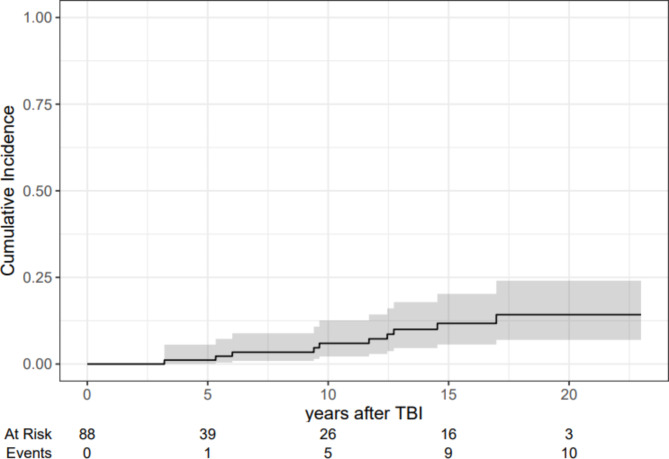
Estimates of the cumulative incidences of secondary solid malignancies with 95% confidence intervals (*n* = 88)

Figure 1 shows the estimates of the cumulative incidences of secondary solid malignancies with 95% confidence intervals treating premature deaths among patients who did not develop secondary solid malignancies as competing risks. The cumulative incidences of secondary solid malignancies were 6% (95%CI, 2–13), 12% (95%CI, 6–20), and 14% (95%CI, 7–24) at 10, 15 and 20 years.

The cumulative incidences of invasive non-SCCs were 4% (95%CI, 1–9), 10% (95%CI, 4–18), and 9% (95%CI, 4–18) at 10, 15 and 20 years. The cumulative incidences of invasive SCCs were 2% (95%CI, 1–8), 2% (95%CI, 1–8), and 5% (95%CI, 1–14) at 10, 15 and 20 years, respectively.

The cumulative incidences of relapse at 5, 10 and 15 years were 43% (95%CI, 33–53), 44% (95%CI, 34–54) and 44% (95%CI, 34–54), while the cumulative incidences of non-relapse mortality (NRM) were 16% (95%CI, 9–24), 18% (95%CI, 11–27) and 18% (95%CI, 11–27), respectively.

### Hazard models for secondary solid malignancies and precancerous lesions and the competing risks of premature deaths

Table 4 shows the cause-specific hazards (HR _CS_) for secondary solid malignancies and precancerous lesions and the competing risks of premature deaths. In the multivariate regression models, older age at TBI translated into lower rates of secondary malignancies/precancerous lesions (HR _cs_ 0.95, 95% CI, 0.90-1.00; *P* = 0.043). The analysis of competing events revealed that older patients showed a trend towards premature deaths (deaths before they could develop secondary malignancies/precancerous lesions, HR _cs_ 1.02, 95%CI, 1.00-1.05; *P* = 0.069). Higher TBI doses (mainly applied in younger patients) translated into reduced rates of secondary malignancies/precancerous lesions (HR _cs_ 0.76, 95% CI, 0.59–0.98; *P* = 0.035) while lacking associations with premature deaths. Chronic GVHD translated into increased rates of deaths before patients could develop secondary malignancies/precancerous lesions (HR _cs_ 8.04, 95%CI, 3.97–16.3; *P* < 0.001).

**Table 4:** Cause-specific hazards (HR _CS_) for secondary malignancies/precancerous lesions and the competing risks of premature deaths (*n* = 88)

	Cause-specific hazard model for secondary malignancies *						Cause-specific hazard model for premature deaths before the development of secondary malignancies *					
	Univariate analysis			Multivariate analysis			Univariate analysis			Multivariate analysis		
	HR_CS_	95%CI	*P*value	HR_CS_	95%CI	*P*value	HR_CS_	95%CI	*P*value	HR_CS_	95%CI	*P*value
Patient age †	0.96	0.91–1.02	0.2	0.95	0.90-1.00	**0.043**	1.03	1.00-1.05	**0.028**	1.02	1.00-1.05	0.069
TBI dose ◊	0.82	0.65–1.03	0.091	0.76	0.59–0.98	**0.035**	0.92	0.83–1.02	0.11	1.00	0.89–1.12	> 0.9
Chronic GVHD #	1.86	0.50–6.91	0.4	1.31	0.31–5.59	0.7	7.85	3.91–15.8	**< 0.001**	8.04	3.97–16.3	**< 0.001**
ATG ‡	0.89	0.27–2.96	0.8	0.67	0.19–2.37	0.5	1.00	0.57–1.77	> 0.9	1.34	0.74–2.42	0.3

* secondary solid malignancies of any histology including precancerous lesions; † patients entered the risk set at the age of TBI; ◊ TBI dose was analyzed metrically; # Chronic graft-versus-host disease (GVHD) requiring systemic immunosuppression was analyzed as time-dependent variable; ‡ Antithymocyte globulin (ATG) was part of GVHD prophylaxis

## Discussion

The present study analyzed the cumulative incidences of secondary solid malignancies and precancerous lesions in AML patients conditioned with TBI before 1st allo-HSCT over 16 years. The cumulative incidences of secondary solid malignancies of any histology including precancerous lesions were 14% and 17% at 15 and 20 years, respectively. We acknowledge a selection bias as RIC containing 4 Gy TBI was applied in older patients with advanced disease status, while younger patients received higher doses of TBI. Multivariate analyses revealed that higher patient age at TBI translated into lower rates of secondary malignancies. The age dependency may be explained by the confounding of competing risks. Older patients mainly receiving lower doses of TBI died more frequently before they could develop treatment-related secondary malignancies. Nevertheless, our results indicate that higher doses of TBI, mainly applied in young patients, were associated with lower rates of secondary malignancies while lacking associations with other causes of mortality. The results partly support radiobiological assumptions and theoretical predictions of carcinogenesis after ionizing radiotherapy suggesting, that the incidence of radiation-induced malignancies increases with increasing radiation dose until a peak is reached and then decreases rapidly [22–24]. Low-dose radiotherapy is not immediately lethal but induces sub-lethal DNA damages and mutations, increasing the risks of carcinogenesis over time. Contrarily, high doses of radiotherapy inducing direct cell death and apoptosis may prevent mutations, and thus carcinogenesis as cell kill becomes the predominant effect. However, the exact dose peak at which cell killing outweighs cell mutation is difficult to predict as molecular, cellular, and tissue-specific factors are integral [22, 23]. Boice et al. [24] analyzed the relationship between radiation doses and leukemia risk after radiotherapy for cancer of the cervix, supporting the radiobiological considerations of carcinogenesis after therapy showing an increased risk for radiation-induced leukemia up to doses of about 4 Gy and a decreased risk at higher doses. Regardless of these radiobiological considerations, we acknowledge the bias of competing risks and the small number of secondary malignancies as confounders in the present analysis.

A reason for making a comparison between studies focusing on the second cancer risk after TBI difficult is the fact that the literature on TBI shows variability in planning and treatment with TBI [25]. Although dose rates of 7.5 cGy/min or less and a twice-daily fractionation are recommended, dose rates and photon energy vary from 2.25 to 37.5 cGy/min and 6 to 25 MV, influencing the risks of secondary solid malignancies after TBI [25]. Furthermore, some studies provide no information about dose rates and the application of lung shielding, which contributes to different toxicity and organ damage after treatment.

Several studies examined the influence of pre-transplantation variables on the risks of second cancers after TBI- and non-TBI-based conditioning [11, 26, 27]. In summary, carcinogenesis after allo-HSCT remains multifactorial, while the number of pretransplant chemotherapy cycles [27], age at exposure, GVHD [12] and its treatment seem to be relevant. Environmental and genetic factors with variabilities in individual susceptibility to DNA-damaging therapies additionally influence the risks of secondary malignancies after allo-HSCT [22, 23]. Scott et al. [28] indicated that clinically photodamaged skin and a history of cutaneous SCC are important risk factors for non-melanoma skin cancer after allo-HSCT, factors not analyzed in the present study. However, the results of the present study are not entirely consistent with other recent studies, which excluded non-melanoma skin cancers or comprised different primary diagnoses. Leisenring et al. [7] analyzed skin and mucosal SCCs and BCCs after TBI- and non-TBI-based conditioning concluding that TBI increases the risks of BCCs but not of SCCs. Modern fractionation with fractionated doses < 13 Gy did not affect the hazards of BCCs in contrast to TBI applied as a single dose and fractionated doses ≥ 13 Gy. Both are no longer recommended as standard [7]. Chronic GVHD and its immunosuppressive therapy seem to increase the risks of SCCs of the skin and mucosa [5, 29], while associations of cGVHD with non-SCCs are less pronounced. The present analysis lacks an association of cGVHD with secondary malignancies (of any histology), which is most likely due to the small patient group. Yet, cGVHD, which represents a main cause of long-term morbidity and mortality, was associated with premature deaths before patients could develop secondary malignancies. Life-long surveillance for secondary malignancies is mandatory for all transplant survivors. Examinations include examinations of the thyroid glands, skin, genitals, and oropharynx [30]. With the help of yearly follow-up examinations, two thyroid cancers were diagnosed in 2 men suffering from cGVHD, which is a known additional risk factor for secondary thyroid cancers [8]. In summary, cutaneous non-melanocytic cancers (BCCs, SCCs) were the most frequent cancer types after TBI, which is in line with recent data demonstrating non-melanocytic skin cancers as the most common cancer types in Germany [31]. The retrospective design and the small number of patients conditioned with TBI limit this study. The study was not powered to analyze risk factors for secondary malignancies of specific anatomical sites and histological subtypes. Moreover, the small number of secondary malignancies apart from non-melanoma skin cancer reduced the generalizability of the results. In addition, the selection bias to treat young patients with TBI and older patients with non-TBI-based regimens, prohibited comparisons of TBI and non-TBI patients regarding secondary malignancies. Nevertheless, the primary strength of the present study is the consistent delivery of modern TBI over 16 years.

## Conclusions

This study indicates a potential inverse relationship between the risk for secondary malignancies and TBI doses applied and is consistent with radiobiological considerations, which assume a decrease in secondary malignancies at high doses. Yet, confounding by competing risks remains a limitation. The use of lower TBI doses in older patients interferes with the age-related increased mortality not caused by secondary malignancies. Therefore, older patients appeared to have lower risks for secondary malignancies than younger patients. This study illustrates the pitfalls of not reporting and considering competing risks in survival data which is relevant in evaluation of newer technologies, such as total marrow irradiation (TMI) and volumetric modulated arc therapy (VMAT) and associated risk assessment for carcinogenesis.

## Data Availability

The data that support the findings of this study are available on request from the corresponding author. The data are not publicly available due to privacy or ethical restrictions.

## Abbreviations

TBI: Total body irradiation
allo-HSCT: Allogeneic hematopoietic stem cell transplantation
AML: Acute myeloid leukemia
SSM: Secondary solid malignancy
IQR: Interquartile range
NRM: Non-relapse mortality
cGVHD: Chronic graft-versus-host disease
MAC: Myeloablative conditioning
RIC: Reduced-intensity conditioning
SCCs: Squamous cell carcinomas
KPS: Karnofsky performance score
HCT-CI: Hematopoietic cell transplantation-comorbidity index
ELN: European LeukemiaNet
ATG: Antithymocyte globulin
MV: Megavoltage
CIF: Cumulative incidence function
CSH: Cause-specific hazard
HR: Hazard ratio
CI: Confidence interval
CR1: First complete remission
PR1: First partial remission
CR2: Second complete remission
FLAMSA: Fludarabine, HD-Ara-C, Amsacrine
MRD: Matched related donor
MUD: Matched unrelated donor
MMRD: Mismatched related donor
MMUD: Mismatched unrelated donor
VIN: Intraepithelial neoplasia of the vagina
CNS: Central nervous system
TMI: Total marrow irradiation
VMAT: Volumetric modulated arc therapy
